# Mammary Tumor Cured with Carbo an

**Published:** 1884-07

**Authors:** J. T. Kent

**Affiliations:** St. Louis


### MAMMARY TUMOR CURED WITH CARBO AN.

PROF. J. T. KENT, A. M., M. D., ST. LOUIS.

Mrs. H. has had several children; she is about thirty-five years old; she has always had much difficulty with all her confinements. The last one was comparatively easy, and yet it was tedious, owing to an elongated cervix. With the first she had an abscess in the mamma (r) and it was badly treated, so that the cicatrix has always been a source of trouble. Preparatory to her last confinement I prepared her as best I could, guided by her symptoms. The child is now some two months old and she is suffering with a hard lump in the right mamma. When I first observed the threatened trouble after the milk began to form, she took Graphites without benefit; also Phytolacca, but only temporary relief followed. The milk mostly dried up, and she now has a nodular lump with retraction of the nipple, and there are lumps in the axilla; she complains of burning and stinging in the lump; and her menstrual flow has come on. She says she has always menstruated during lactation. The flow is dark and clotted; when she goes to sleep she perspires freely; she seems greatly prostrated after a moderate loss of menstrua; she is somewhat cachectic.

For a choice of remedy we might arrange:

Burning in Mammæ.—Apis, *Bell.*, Calc., *Carbo an.*, *Iod.*, Led., Mez., Selen., Laur., *Phos.*, Lyc., *Tarent-c.*

Stinging in Mammæ.—*Apis*, *Berb.*, *Carbo an.*, Con., Kreosote, Graph., Gratiola, Ind., Iodine, Kali c., Laur., *Lyc.*, *Murex*, Nat-m., *Phos.*, Rheum, Sang., Sepia.

Nodosities in Mammæ.—Bell., Carbo an., Colocy., Con., Graph., Lyc., Nit-ac., Sil.

Cancer of Mammæ.—(Minton), Bell., Carbo an., Coloc., Con., Graph., Lyc., Nit-ac., Sil.

Perspiration during Sleep.—*Carbo-an.*, Cicuta, Chin., Dros., Euph., Ferr., Jatroph., Merc., Nux, Phos., *Puls.*, Selen., Thuja.

Great Exhaustion after Menses.—Alum., Carbo an., Chin., Ipecac., Phos.

Menses during Lactation.—Calc., Sil.

Neither of the last remedies correspond to the balance of the symptoms. But it will be seen that Carbo-an. and Phos. cover the case, and the menstrual flow, which is *dark* and *clotted*, is not so characteristic of Phos. as Carbo an. The exhaustion after the flow is more marked in Carbo-an. than in Phos., though both have it in a marked degree.

"*The flow weakens her; she can hardly speak; blood dark;* (*Guiding Symptons*) under Carbo an. Carbo an.$^{3m}$, one dose dry, was administered. Four weeks, burning and stinging all gone; glands in axilla nearly gone. After the dose the cutting pains became worse for a few days. Medicine repeated in thirty-nine days. The lump has disappeared.

